# Atypical Presentations and Molecular Diagnosis of Ocular Bartonellosis

**DOI:** 10.3390/ijms262110421

**Published:** 2025-10-27

**Authors:** Munirah Alafaleq, Christine Fardeau

**Affiliations:** 1 Ophthalmology Department, King Fahd Hospital of the University, Imam Abdulrahman Bin Faisal University, Dammam 31441, Saudi Arabia; maalafaleq@iau.edu.sa; 2 Ophthalmology Department, Reference Center for Rare Diseases, Pitié-Salpétrière Hospital, University Paris Sorbonne, 47-83 Boulevard de l’Hôpital, 75013 Paris, France; 3 Department of Ophthalmology, Hôpital Lariboisière, AP-HP, Université de Paris, 75013 Paris, France

**Keywords:** Bartonella, neuroretinitis, cat scratch disease, uveitis, choroiditis, optic disc swelling, imaging

## Abstract

To describe unusual findings and management of neuroretinitis in patients with cat scratch disease (CSD), their functional outcome after a case-oriented treatment was anaylsed, and the current literature was reviewed. A retrospective monocentric case series and a literature review. Review of medical records, multimodal imaging, and literature review. Five patients (four females and one male) with a mean age of 29.75 years (range: 11–71 years) had unusual findings of ocular bartonellosis, including inner retinitis, focal choroiditis, retinal microaneurysms, and bilateral sectorial optic nerve swelling. *Bartonella*-related ocular infections were not limited to the posterior segment of the eye. Molecular tests, such as polymerase chain reaction (PCR), showed that elevated markers of IgG titers were used and were positive in the aqueous humour of one patient. Reference to the use of intravitreal treatment in one of the cases was useful. Case-oriented management is associated with improvement in visual acuity, retinal, and choroidal lesions. The range of ocular signs of Bartonella infection could be extended. Molecular tests, such as PCR, are useful diagnostic approaches in the diagnosis of posterior uveitis. Treatment could require intravitreal antibiotic injections in unusual ocular bartonellosis.

## 1. Introduction

Cat scratch disease (CSD) is characterised by lymphadenopathy, fever, skin lesions and neuroretinitis in immunocompetent individuals following a cat scratch or bite [1,2]. *Bartonella henselae* (*B. henselae*) (formely Rochalimaea) is the primary bacterial agent responsible for CSD and ocular bartonellosis [3]. Neuroretinitis is characterised by optic disc swelling and a macula star, which was called Leber’s idiopathic stellate neuroretinitis [4]. The development of an indirect fluorescence antibody test allowed the detection of antibodies directed against *B. henselae* in patients with CSD [3], along with molecular tests, such as polymerase chain reaction (PCR) [5,6,7,8,9]. Neuroretinitis due to B.henselae has also been reported without CSD [10] and can affect both adults and children [11,12]. Different atypical ocular and systemic manifestations associated with *B. henselae* have been reported [13,14,15,16,17]. The rare documentation of an atypical case of bartonellosis rendered the management of ocular bartonellosis challenging, as there is no consensus [18,19,20,21,22]. The current study aimed to describe the diagnosis of atypical ocular bartonellosis, their multimodal imaging, the case-oriented approach of their management in our department, and to review the literature concerning bartonella-related intraocular infections.

## 2. Results (Table 1)

Five patients (four females and one male) with a mean age of 29.75 years (range: 11–71 years) presented unusual signs of neuroretinitis, including inner retinitis, focal choroiditis, retinal microaneurysms, and bilateral sectorial optic nerve swelling. The five patients were found to be positive for Bartonella either after serology or intraocular fluid sample PCR. Case-oriented management was associated with improvement in visual acuity, retinal, and choroidal lesions.

**Table 1:** Summary of Clinical findings and Management of patients with Atypical presentation of Ocular Bartonellosis.

Case	Age/Gender	Symptoms	Diagnostic Findings	Treatment	Outcome
1	20 M	Fever, ↓ VA RE, bilateral optic disc swelling, scotomas	Bartonella IgG+, choroiditis, retinitis, macular star, CSF mild pleocytosis	Ciprofloxacin + Azithromycin (allergic to sulfa)	Full recovery (20/20 both eyes)
2	17 F	Fever, ↓ VA RE, central scotoma	Bartonella IgG+, optic disc swelling, macular star, focal haemorrhage, focal choroiditis	Rifampicin + TMP-SMX	Full recovery (20/20)
3	71 F	Bilateral ↓ VA, resistant posterior uveitis	Bartonella DNA (PCR+, vitreous), microaneurysms, placoid exudates, choroidal hypoperfusion	Intravitreal gentamicin + ciprofloxacin → Rifampicin + TMP-SMX + low-dose steroids	Partial recovery (20/63 RE, 20/200 LE, fibrosis)
4	11 F	↓ VA RE, bilateral sectorial optic nerve swelling	Bartonella IgG+/IgM+, bilateral macular edoema (OCT), normal MRI/LP	Azithromycin (age contraindicated quinolones)	Full recovery (20/20 both eyes)
5	30 F	Painful ↓ VA LE, papillary edoema, serous RD	Bartonella IgG+, focal choroiditis, negative systemic workup	Rifampicin + TMP-SMX (10 days)	Full recovery (20/20 in 8 weeks)

↓ Means decreased, and VA is the Visual aquity.

### 2.1. Case 1 (Figure 1)

A 20-year-old male consulted after a 3-day history of decreased visual acuity in his right eye. He was febrile for 6 days with a temperature of 40 °C. He had a kitten and had been scratched on his hand about one month before. His vision was reduced to “counting fingers” in his right eye and to 20/20 in his left eye. No inflammatory cells were found in the anterior chamber or vitreous in both eyes. Flare was 9.1 and 2.6 photons/ms in RE and LE, respectively (normal value < 8 ph/ms). The significant findings were optic nerve swelling in both eyes, superficial haemorrhage at the edge of the optic nerve, round, deep yellow active choroidal lesions in the posterior pole and the periphery, and a superficial retinitis lesion.

Blood tests revealed an increase in CRP to 103 mg/dL with polymorphonucleocyte count at 4700/mm^3^ and lymphocyte count at 450/mm^3^. Bartonella serology showed positive results with an IgG titer at 1/100 against *B. henselae*. Results of the tuberculin skin test were negative. Other infectious and autoimmune workup was negative. Aqueous humour sampling was tested for herpes virus, toxoplasmosis PCR, and was negative. The brain computed tomography (CT) scan was normal. Lumbar puncture showed 25 leukocytes/mm^3^ with interferon A level less than 2 units/mL and protein level at 45 g/L (15–45). Allergic to sulfamides, he received 250 mg of ciprofloxacin twice a day associated with 250 mg of azithromycin twice a day for three weeks, with a complete recovery of visual acuity to 20/20 in both eyes and visual field.

**Figure 1:**
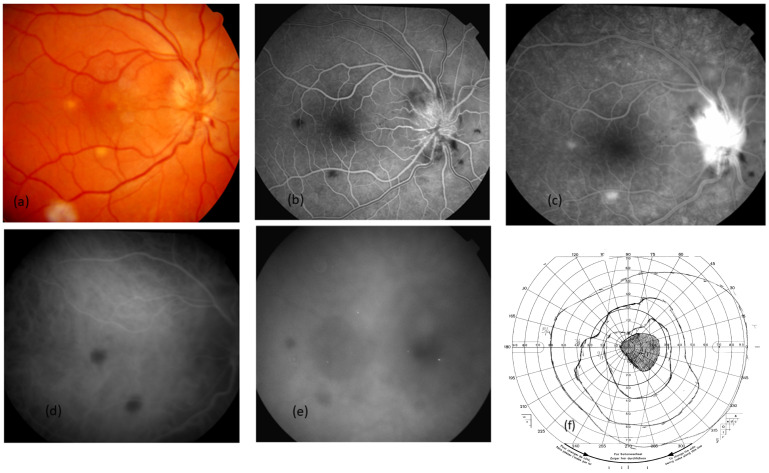
Case 1. A 20-year-old male, febrile for 6 days with B-henselae positive serology, presented with decreased VA in his RE. (**a**) RE Colour photograph showing swollen disc with superficial haemorrhages, two deep nodular whitish lesions corresponding to focal choroiditis and a whitish retinitis lesion along the temporal inferior vein. A few days later, macular exudates started to appear. (**b**) RE FA early arteriovenous showing dilated vessels at the optic disc and hypofluorescent round focal choroiditis in the posterior pole. (**c**) RE FA late phase showing hyperfluorescence at the optic disc and focal choroiditis in the posterior pole. (**d**) RE ICGA early phase showing hypofluorescent round lesions corresponding to focal choroiditis. (**e**) RE ICGA late phase showing hypofluorescent centromacular and peripapillary lesions corresponding to serous retinal detachment. (**f**) Visual field Goldmann showing coecocentral scotoma corresponding to the hypofluorescent lesions seen in ICGA late phase.

### 2.2. Case 2 (Figure 2)

**Figure 2:**
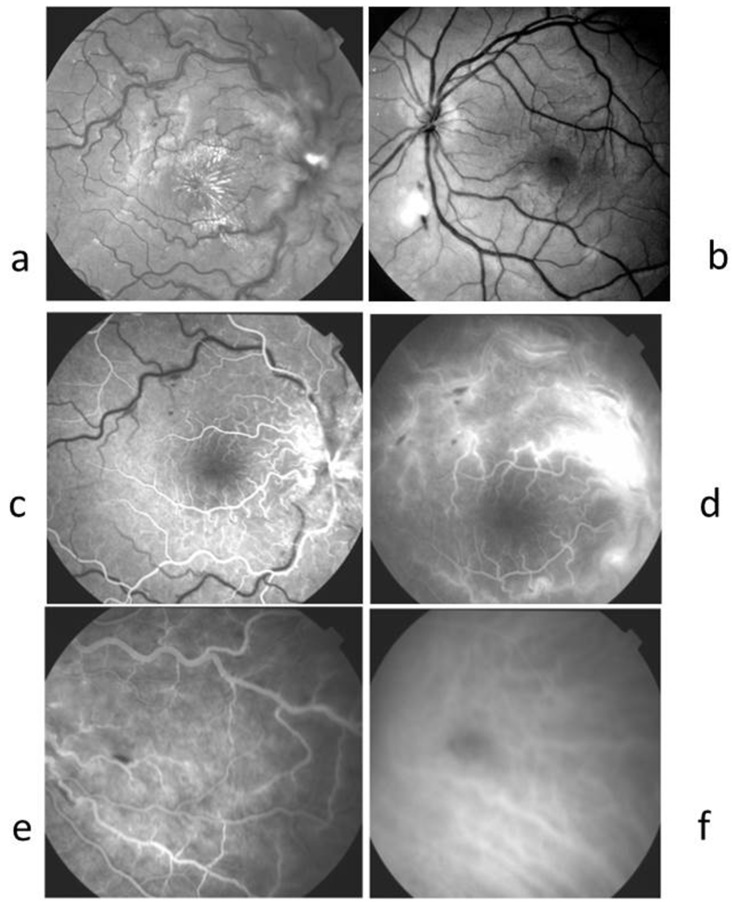
Case 2. A 17-year-old female, febrile for 1 week, had decreased vision in her right eye for 3 weeks associated with a central scotoma. (**a**) Red-free photography RE showing papilledema, stellar macular exudates. (**b**) Red-free photography LE showing superficial haemorrhages and whitish focal retinitis. (**c**) RE FA at the early phase showed a delayed venous filling (**d**) FA late phase showed leakage from retinal and papillary dilated capillaries. (**e**) Nasal periphery in FA of the fundus showed a focal superficial haemorrhage. (**f**) ICGA RE nasal periphery showing a hypofluorescent round area, suggesting focal choroiditis in the same area than retinal superficial haemorrhage.

A 17-year-old female consulted for decreased vision in her right eye for 3 weeks associated with a central scotoma. She was febrile for 1 week. She had a kitten and had been scratched two weeks before symptom initiation. Her visual acuity was 20/32 in the RE and 20/20 in the LE. No signs of inflammatory cells were found in the anterior and posterior segments in both eyes. An optic disc swelling associated with superficial haemorrhage, venous dilation, and a macular exudate star was observed in RE. Goldmann visual field showed centro-caecal scotoma in the RE. The LE showed a normal visual field. Findings in FA and ICGA are described in the legends of Figure 2. Blood tests showed an increase in CRP of 14 mg/dL (1–5) with a WBC count of 13,000/mm^3^. *Bartonella henselae* was positive with an IgG titer at 1/64, while other infectious and autoimmune tests were negative. Aqueous humour sampling was tested for herpes virus and toxoplasmosis PCR and was found to be negative. The treatment included 10 mg/kg of rifampicin and sulfamethoxazole trimethoprim for 3 weeks, and the vision in the right eye returned to 20/20 with normalisation of the visual field.

### 2.3. Case 3 (Figure 3)

**Figure 3:**
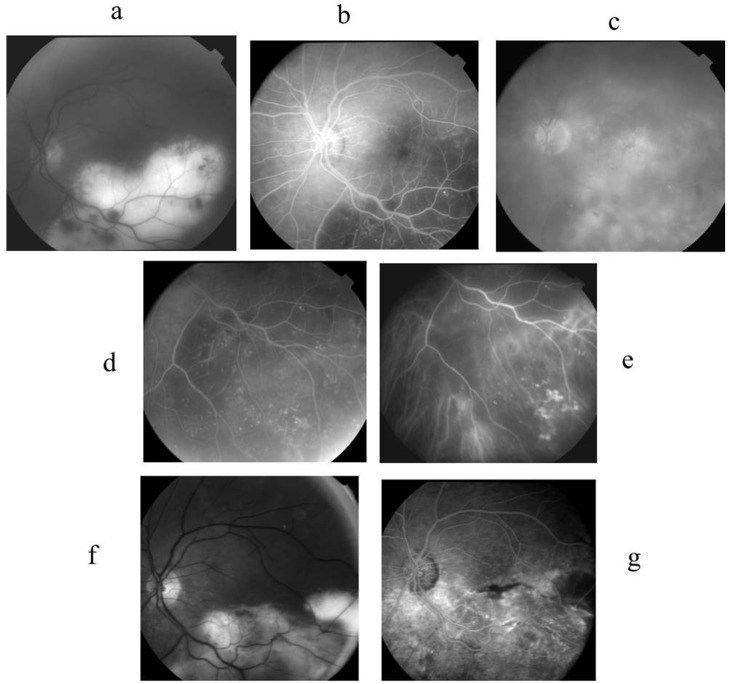
Case 3. A 71-year-old female presented with a visual loss to 20/100 RE and 6/240 LE due to a bilateral posterior uveitis resistant to high doses of steroid treatment. The RE showed optic disc area leakage in FA and a coecocentral scotoma in the Goldman visual field. The LE showed the following: (**a**) Red-free photograph showing the extent of placoid deep exudates with superficial haemorrhages and retinal vessel anomalies. (**b**) FA early phase showing an enlargement of the hypofluorescent area due to both a shadow effect of placoid exudates and a hypoperfusion at the choroid. (**c**) FA late phase showing hyperfluorescent cysts of macular edoema and diffuse leakage in the posterior pole and inferior periphery. (**d**) FA inferior periphery showing hypofluorescent area corresponding to exsudates masking area and hyperfluorescent aneurysms. (**e**) ICGA inferior periphery showing choroidal hypofluorescence and well-seen retinal aneurysms. (**f**) Red-free photography showing reduced exudates leaving intraretinal fibrosis after 2 months of antibiotic treatment, (**g**) FA, 3 min showed macular pigmentary migration, inhomogeneous choroidal and retinal fluorescence suggesting subretinal fibrosis, and a few aneurysms persisted after 2 months of antibiotic treatment.

A 71-year-old female was referred to our centre after treatment with 20 mg of oral prednisolone for 6 months, with no improvement in her bilateral posterior uveitis and reduction in visual acuity to 20/100 in the right eye and 6/240 in the left eye. She had no previous history of a cat scratch. Ocular examination of the right eye revealed a moderate vitritis with optic disc swelling. In the left eye, there were granulomatous precipitates in the cornea with retinal exudate, placoid lesions in the inferior temporal quadrant with numerous microaneurysms, and cystoid macula oedema. Findings in FA and ICGA are described in the legends of Figure 3. Goldman visual field showed a right centro-caecal scotoma and a left superonasal visual field defect, together with a centro-caecal scotoma. Flare values were 27.3 and 35.9 in RLE.

Blood tests showed a slight increase in leukocytes to 11,000/mm^3^ (4000–10,000). ACE level was twice the normal level, while lysozyme was within normal range. Other infectious tests were negative. Lumbar puncture revealed 6 nuclear cells/mm^3^ with no proteins. Aqueous humour sampling was tested for interleukin (IL)10/6, herpes virus, and toxoplasmosis PCR; all were negative. A CT scan showed mediastinal lymph nodes, and the diagnosis of possible sarcoidosis was suggested. She received a bolus of 3 doses of intravenous methylprednisolone. However, her vision deteriorated despite long-term prednisone treatment, for she underwent diagnostic vitrectomy in her left eye. Vitreous cytology analysis showed numerous lymphocytes and macrophages. All lymphocytes were CD3^+^, CD4^+^, CD8^−^, and CD19^−^, suggesting an infectious process. PCR for the herpes virus and toxoplasmosis was negative. Toxocariasis and fungal infections were ruled out. Periodic-Acid Schiff test was negative, IL-10 level was 14 pg/mL (0–8). A light Cycler PCR targeting Bartonella spp. was positive, and Bartonella Quintana DNA was identified after PCR amplification and sequencing of this fragment, as previously described [23,24]. Following diagnosis, she received intravitreal gentamicin 1 mg in the left eye in combination with 1 g/day of oral ciprofloxacin for 3 weeks. Thereafter, she received 5 mg/kg of oral rifampicin and sulfamethoxazole trimethoprim combined with 7 mg/day of prednisone for 2 months. For these two months of treatment, quinolones were avoided because of the risk of developing tendinitis. At her last visit, the visual acuity was 20/63 in her right eye and 20/200 in her left eye. Microaneurysms disappeared, and consequently, exudates disappeared too. 

### 2.4. Case 4 (Figure 4)

**Figure 4:**
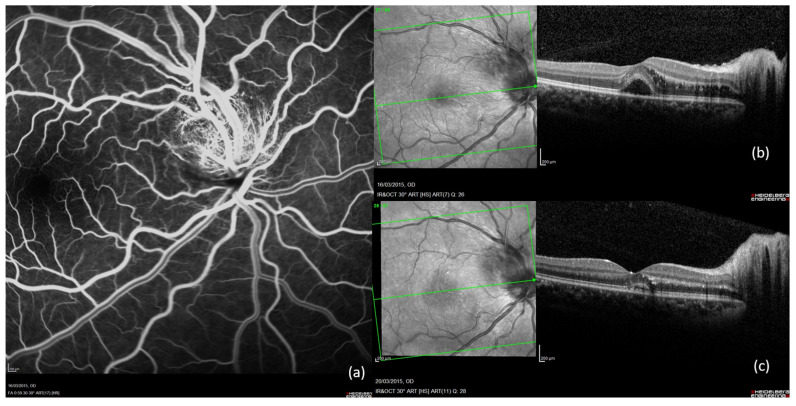
Case 4. 11-year-old female with decreased visual acuity of RE 20/70, LE 20/20 presenting a bilateral sectorial optic nerve swelling. (**a**) RE late FA showing a superior optic nerve swelling without pallor or signs of hyperaemia. (**b**) RE initial SD-OCT showing macular oedema. (**c**) RE SD-OCT after one month of treatment, showing numerous hyperreflective exudates well visible in the outer plexiform layer after resorption of macular oedema.

An 11-year-old female consulted for decreased visual acuity to 20/70 in the right eye and 20/20 in the left eye. She had been scratched by her cat many times. On ocular examination, no inflammatory cells were found in the anterior chamber or vitreous. Fundus showed sectorial swelling of the optic nerve head in both eyes without pallor or signs of hyperaemia. Initial SD-OCT of her RE macula showed retrofoveal subretinal liquid accumulation, while LE macula showed no exudation signs. Findings in FA in RE are described in the legends of Figure 4. Goldmann visual field revealed a central scotoma in the right eye and a centrocecal scotoma in the left eye. Colour vision tested with the Farnsworth-Munsell 15-hue was not affected. The CRP level was increased to 33 mg/dL (>5 mg/dL). Serum analysis revealed a positive *B. henselae* IgG serology with positivity for IgM antibodies. Other serology tests were negative. Aqueous humour sampling was tested for herpes virus and toxoplasmosis PCR, and was determined as negative. The head magnetic resonance imaging was normal. Analysis of the lumbar puncture was normal. Because of the age of 11, quinolones were avoided, a systemic antibiotic therapy was initiated with 250 mg of azithromycin daily for 4 weeks. Stellate exudates visible on fundus examination appeared one week after partial resorption of the macular oedema of the right eye under antibiotics. During the following month, a complete resolution of the macular oedema and stellate exudates was observed, and the patient recovered a full visual acuity to 20/20 in both eyes. This case was atypical because the optic nerve swelling was sectorial in both eyes.

### 2.5. Case 5 (Figure 5)

A thirty-year-old female presented in the emergency department because of progressive LE visual loss for 2 weeks. LE was red and painful. History revealed a bite by a rat and a cat scratch. VA was 20/20 RE and 20/100. LE showed papillary oedema, active round yellow deep lesions in the posterior pole, serous retinal detachment in the macula centre, and in the inferior area to the optic nerve, while RE was normal. No fever or adenopathy. Wide infectious and auto-immune workup was negative except for Bartonellae hansalae showing IgG up to 1/128. Aqueous humour sampling was tested for herpes virus, toxoplasmosis PCR, and was negative. Treatment included 10 mg/kg of rifampicin and sulfamethoxazole trimethoprim for 10 days. Full recovery of VA up to 20/20 achieved in 8 weeks.

**Figure 5:**
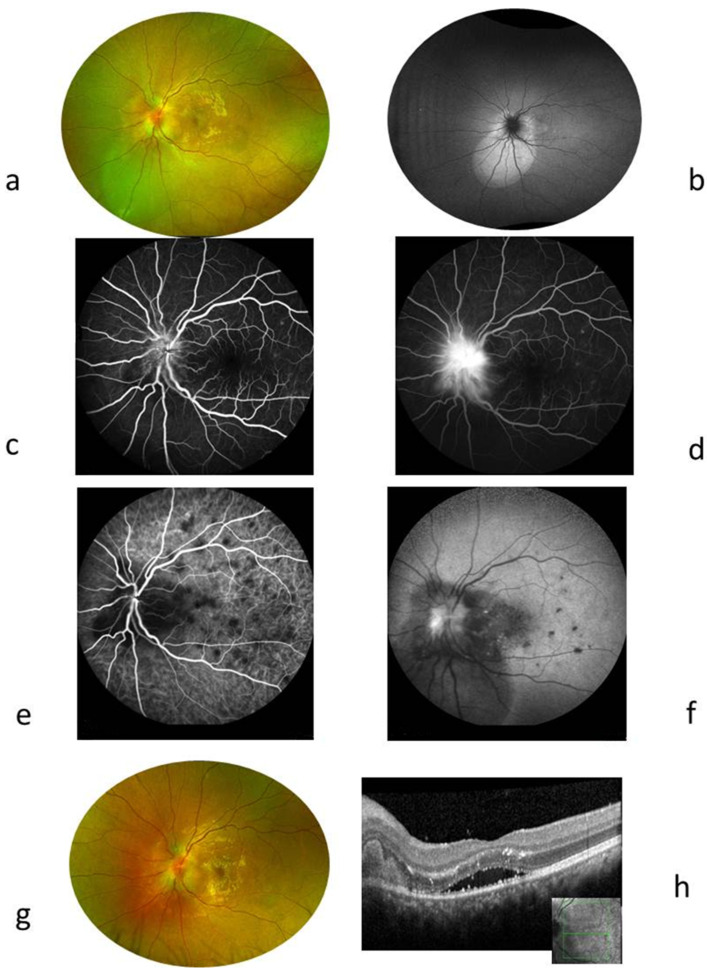
Case 5: F. 30 years old, complaining of visual loss in LE to 20/100. (**a**) Fundus showed serous retinal detachment in the macula centre and the inferior area to the optic nerve head, round deep retinal lesions, and papillary oedema. (**b**) Autofluorescence showed hyperautofluorescent serous retinal detachment at the edge of the optic nerve. (**c**,**d**) FA showing the hyperfluorescent papillary edoema in the late phase. (**e**,**f**) ICGA showing numerous hypofluorescent round lesions corresponding to focal choroiditis that some were erased in the late phase. Four days later, stellar exudates were visible in the fundus shown in (**g**,**h**) OCT showed hyperreflective juxta papillary material and hyperreflective exudates mainly located in the outer plexiform layer, associated with centromacular serous retinal detachment.

## 3. Discussion

Neuroretinitis due to CSD typically includes optic disc swelling and macular exudate star, usually unilateral and self-limited, initially described as Leber’s neuroretinitis and well defined by Gass as an exudative optic neuroretinitis with transudation into an apparently normal macula [25]. Serologic tests for *B. henselae* infection have shown that CSD is the most common syndrome associated with neuroretinitis [26].

In addition to the characteristic optic nerve swelling and macular exudates, our 5 cases presented other signs, including phlebitis, retinitis, choroiditis, choroidal hypoperfusion, and bilateral sectorial nerve swelling. Rare signs have been described in CSD, such as branch retinal artery occlusion, branch retinal vein occlusion, and retinal phlebitis [15,27,28,29]. Isolated chronic vitritis [30] and pars planitis [31] have been described in a few patients with active *B. henselae* infection. Acute multifocal inner retinitis in white dot syndrome has been described in many patients with disseminated ocular CSD [26,27,29,32]. Retinitis with dense white retinal infiltrates and haemorrhages has been reported by Warren et al. in a patient with AIDS; a biopsy revealed retinal bacillary angiomatosis, and *B. henselae* infection was confirmed by PCR [33]. Disciform keratitis, neovascular glaucoma, and orbital abscess were also reported [34,35,36]. Diffuse choroidal thickening with late subretinal leakage on FA could suggest Vogt–Koyanagi–Harada disease [37].

In the current study, multiple nodular choroiditis were found in all cases, associated with either inner retinitis, retinal microaneurysms, or sectorial papilledema. Herpes virus, toxoplasmosis, Toxocariasis, and fungal infections were ruled out after a full workup, including blood associated with aqueous humour samplings.

Bartonella species have a propensity to invade the vascular endothelium [38] which could explain these clinical findings. Following antibiotic therapy, visual recovery was observed in patients with peripapillary angiomatosis with exudative peripapillary retinal detachments [39,40].

Although usually unilateral, bilateral neuretinitis in CSD may occur, as shown in our third case [26,32]. The blood serology for Bartonella was positive in five cases and one case showed positive PCR results for Bartonella Quintana in a vitreous sample. PCR is useful in differentiating infectious from non-infectious causes of posterior uveitis. Furthermore, Intraocular PCR can detect fastidious bacteria missed by serology or routine labs, as in our Case 3 where *Bartonella quintana* was confirmed. PCR is especially useful when corticosteroid use obscures aetiology or when masquerade syndromes are suspected.

The etiologic evaluation of uveitis could be unsuccessful when non-invasive methods are used. Among 1321 ocular samples previously studied, an infection was diagnosed in 11% of samples using a standardised protocol for laboratory investigations, including universal PCR-based detection of any bacteria, fungi, specific PCR-based detection of fastidious (difficult-to-grow), herpes viruses, and culture of vitreous fluid. Half of the positive results were linked to fastidious bacteria, including Bartonella species [41].

Indirect immunofluorescence test for the detection of serum anti-*B. henselae* antibodies are the most widely accepted detection method in CSD. However, the specificity and sensitivity for Bartonella species have been improved by the development of PCR-based tests, including sequencing and Light Cycler assay. The diagnosis of bartonellosis is based on the clinical presentation of symptoms and an evaluation of risk of exposure. However, the non-specific symptoms necessitated specific diagnostic confirmation tests, such as the detection of specific IgG or DNA. The PCR targeted the gene *rpoB* was performed on the biopsy or puncture, permitting the detection of Bartonellae. LightCycler technology combines rapid-cycle polymerase chain reaction with real-time fluorescent monitoring and melting curve analysis. Since its introduction in 1997, it has been used in many areas of molecular pathology, including oncology (solid tumours and hematopathology), inherited disease, and infectious disease [42].

CSD is known as a self-limiting disease, but significant visual complications in our atypical cases lead to the prescription of specific antibiotic treatment in informed patients, as previously suggested [26]. There is no consensus on the choice of antibiotics [18,19,20,21,22].

Conventional treatment includes doxycycline which shows a very low penetration through the blood–brain barrier. The current use of 10 mg/kg of rifampicin and sulfamethoxazole trimethoprim b.d. for 3 weeks has shown good results in previous studies, as well as in our second, third, and fifth cases [43]. Chronic *B. quintana* bacteremia is optimally treated with doxycyclin plus gentamicin [43]. However, oral doxycycline and gentamycin had shown a low level of intra-ocular penetration [44,45]. Ciprofloxacin, from the quinolone group, can cross the blood-ocular barrier and has shown efficiency in treating Bartonella uveitis as seen in our first case [31,32,44].

The aminoglycosides, such as gentamycin, have shown a low level of intraocular penetration because of high molecular weight and low lipid solubility, and so intravitreal injection is needed to treat the retina, as used in our third case [45]. Azithromycin has shown a high ocular penetration level and a clinical efficiency, as shown in our first and fourth cases [46].

So, to treat severe intra-ocular complications of Bartonella infection, a balance between benefit and risk could be performed using ciprofloxacine + sulfamethoxazole trimethoprim, which could be switched to azithromycin in case of sulfamide allergy in adults. Moreover, in case of refractory retinal exsudation, an associated gentamicin intravitreal injection could be discussed, while macular toxicity is well known and could be reversible for short-term exposure to gentamicin [47]. Azithromycin associated with sulfamethoxazole trimethoprim could be suggested in case of severe ocular complications in children.

Treatment regimens were individualised—ciprofloxacin + azithromycin for sulfa allergy (Case 1), rifampicin + TMP-SMX as standard (Cases 2 and 5), intravitreal gentamicin plus systemic therapy for PCR-confirmed, steroid-refractory disease (Case 3), and azithromycin in a child (Case 4). These examples illustrate how age, allergy profile, and disease severity guide antibiotic choice.

These examples illustrate how age, allergy profile, and disease severity guide anti-biotic choice.

## 4. Materials and Methods

### 4.1. Study Design

A retrospective case series study of medical records of 1854 patients diagnosed with uveitis seen between 1 January 2011 and 31 December 2024 in a tertiary reference centre, showing Bartonella positive on ocular or blood samples.

### 4.2. Exclusion Criteria

Patients with typical ocular bartonellosis, including optic nerve swelling and macular star, were excluded.

### 4.3. Diagnostic Definitions

Positive Bartonella diagnosis: An IgM titer of 1:16 or higher shows acute disease, with a 3-month duration of detection in 50% of patients. An IgG titer higher than 1:256 is considered evidence of current or past Bartonella infection.

### 4.4. Ethics and Consent

Research followed the Tenets of the Declaration of Helsinki. Informed consent is signed by patients before aqueous humour, vitreous sampling, or lumbar puncture.

### 4.5. Ophthalmic and Wide Workup Assessment

Patients were assessed by spectral domain optical coherence tomography (SD-OCT), fluorescein, and indocyanin green retinal angiography (FA and ICGA) after full clinical examination. A wide workup to rule out infectious causes—in particular tuberculosis, autoimmune diseases, sarcoidosis, and tumoral causes intravitreal lymphoma—was performed. It included a full blood count, ionogram, sedimentation rate, C-reactive protein (CRP), syphilis, human deficiency virus (HIV), Lyme disease, Toxocariasis, Rickettsia, Brucellosis, bartonellosis serologies, antinuclear antibodies, quantiferon test, tuberculin skin test, chest tomography, and cerebral imaging. All patients were examined by an internist physician.

On admission, the presence of bilateral papilledema in 2 patients raised the urgency to perform cerebral imaging, followed by lumbar puncture to measure the pressure of the Cerebrospinal Fluid (CSF), cytology, and electrophoresis. All cases were discussed in multidisciplinary meetings before treatment. Analysis of the current literature concerning Bartonella-related intraocular infections was also carried out.

### 4.6. Molecular Analysis

Aqueous humour sampling was tested for herpes virus, toxoplasmosis and bartonellosis by molecular tests, such as polymerase chain reaction PCR. In an elderly patient Interleukin 10/6 was analysed in addition to rule out a non-Hodgkin intraocular lymphoma mimicking uveitis [48].

## 5. Conclusions

Bartonella infection clinical spectrum seems compatible with an atypical presentation of neuroretinitis. SD-OCT, FA, and ICGA are helpful and complementary imaging tools in the diagnosis decision-making. Intraocular puncture or biopsy is a useful diagnostic approach in the diagnosis of posterior uveitis whose aetiology remains unknown. PCR testing from intraocular fluids could be considered in cases of posterior uveitis with unknown aetiology. Different approaches to antibiotic treatment seem associated with an improvement in visual acuity and retinal and choroidal lesions. A balance between benefit and risk and discussion with a multidisciplinary team is the gold standard in the management of unusual cases of ocular bartonellosis. Multicenter studies or prospective registries can be considered to confirm these findings.

## Data Availability

The original contributions presented in this study are included in the article. Further inquiries can be directed to the corresponding author(s).

## Abbreviations

*B. henselae*	*Bartonella henselae*
SD-OCT	Spectral Domain Optical Coherence Tomography
FA	Fluorescein Angiography
ICGA	Indocyanine Green Angiography
CSD	Cat scratch disease
CRP	C-reactive protein
WBC	White blood cell
HIV	Human immunodeficiency virus
ACE	Angiotensin conversion enzyme
CT	Computed tomography scan
VDRL	Venereal Disease Research Laboratory
